# Perspectives on promoting healthy pregnancies

**DOI:** 10.4069/whn.2024.03.13.1

**Published:** 2024-03-29

**Authors:** Hae Won Kim

**Affiliations:** The Research Institute of Nursing Science, SNU Center for Human-Caring Nurse Leaders for the Future by Brain Korea 21, Seoul National University College of Nursing, Seoul, Korea

## Nursing challenges for healthy pregnancies

The role of nurses with specialized competencies in the care of women with high-risk pregnancies has been emphasized by the Maternal Fetal Intensive Care Unit support project [1]. The Korean government has increased its financial support for medical expenses in this population. Initially, in 2015, governmental policy covered three conditions: preterm labor, placental abruption, and severe pre-eclampsia. By 2019, the policy had expanded to cover a total of 19 conditions, including hypertension, multiple pregnancy, and gestational diabetes [1]. However, despite considerable national health expenditures on the management of high-risk pregnancies, disease-focused clinical approaches have only marginally improved the outcomes of these pregnancies.

The medical definition of a high-risk pregnancy is one with a greater likelihood of a poor outcome compared to a typical pregnancy. However, the enumeration of all specific risk factors is challenging due to the numerous elements that can affect pregnancy outcomes, including women’s lifestyle, prepregnancy health, family history, history of pregnancy, and social and environmental factors [2]. In 2012, the Korean Society of Maternal Fetal Medicine introduced the concept of risk factors for high-risk pregnancy as an academic term in Korea. By 2016, the classification of high-risk pregnancy included obstetric risk factors, medical risk factors, physical risk factors, and current pregnancy risk factors. Each category of risk factors was further classified into three levels of severity: mild (grade I), moderate (grade II), and severe (grade III) [2]. Thus, medical definitions and explanations related to high-risk pregnancy are complex and may be perplexing for the layperson.

The Korean government has recently implemented policies to support couples with infertility, provide fetal deformity screening, and cover medical expenses before birth. After childbirth, maternal and newborn health care services, maternity supplies, childbirth incentives, and financial support for postpartum care are available in the local community [3]. However, it remains necessary to increase social awareness of the importance of healthy pregnancies and promote the practices essential for maintaining healthy pregnancies at the individual or group level.

Promoting healthy pregnancies is a concept that extends far beyond the clinical management of high-risk pregnancies, encompassing financial and medical support to pregnant and postpartum women. The objective of nursing in this context is not solely to reduce the diagnoses of high-risk pregnancies but also to prepare for, promote, and maintain healthy pregnancies. Therefore, this article was designed to explore the expanded roles of nursing in promoting healthy pregnancies by presenting two perspectives on the subject, along with conceptual and empirical examples.

## Perspective 1

### Gender equality and the life cycle approach: developing customized nursing care for healthy pregnancies

#### Philosophy of healthy pregnancies: acknowledging the equal rights and responsibilities of men and women

Everyone possesses the right to health, including the right to a healthy pregnancy. To uphold this latter right, the responsibilities assigned to men and women must be equal.

Preconception health and health care, as outlined by the U.S. Centers for Disease Control and Prevention [4], include men as key contributors and incorporate them in its recommendations. Preconception health emphasizes proactive measures to safeguard the health of a future child and encompasses the broader maintenance of overall health, not just in preparation for pregnancy but as part of a lifelong commitment to healthy living. Consequently, preconception health care (PCC) varies according to each person’s specific needs, yet it remains crucial for both women and men [4]. Therefore, a gender equality perspective is integral, and this should be emphasized in the promotion of healthy pregnancies.

#### Customized life cycle approach: supporting healthy pregnancies from adolescence

A reproductive life plan (RLP) is a patient-centered approach that focuses on an individual’s reproductive preferences, whether well-defined or uncertain. The RLP process enables both women and men to consider how reproduction fits within the broader context of their lives [5]. RLP enables reflection on desires regarding pregnancy and facilitates the establishment of goals in the context of the entire lifespan [6]. This approach promotes the planning of a healthy pregnancy and childbirth, tailored to each person’s unique circumstances and life stage. Adolescence is a key period for preparing for a healthy pregnancy. This outlook represents a shift from focusing solely on the window immediately before conception [7]. In particular, teenage pregnancy is recognized as a high-risk category, and research has demonstrated a significant correlation between the lack of prenatal care among pregnant Korean teenagers and the incidence of premature birth [8]. Despite this, Korea currently has no national consensus or established guidelines on the necessary preparations for healthy pregnancies during adolescence.

#### Developing nursing knowledge on healthy pregnancies tailored to the target population

In the Netherlands, promoting healthy pregnancies is a high priority, and the nationwide initiative Healthy Pregnancy 4 All (HP4ALL) has been underway since 2011 [9]. PCC is considered an essential component of the medical care system to improve perinatal outcomes [9]. PCC includes individual consultations focused on risk assessment and risk management, as well as follow-up appointments to evaluate adherence to the management plan. The primary outcomes of PCC consultations include successful behavioral changes, such as taking folic acid supplements, quitting smoking, and abstaining from alcohol and illicit substance use [9]. In the United States, the Advanced Preconception Wellness Recommendations, released in 2016, represent a national consensus on prepregnancy health and management. These recommendations cover a range of topics, including planned pregnancy, access to care, vitamin intake, smoking cessation, and avoiding exposure to teratogenic substances [10]. Building on this, the concept of healthy pregnancy preparation behavior (HPPB) was operationalized in a recent study [7] to include (1) proper contraceptive use; (2) planning for pregnancy and childbirth in advance; (3) abstaining from sexual activity until adulthood; (4) avoiding binge drinking; (5) refraining from smoking; (6) preventing and managing sexually transmitted infections; (7) maintaining a healthy weight and engaging in physical activity; (8) being cautious of harmful chemicals or environmental substances; (9) seeking help in cases of verbal, physical, or sexual violence; (10) maintaining mental health; and (11) getting vaccinated as needed. Additionally, the concept of gender equality attitudes related to pregnancy and birth was incorporated, and 10 questions were formulated to explore the roles and responsibilities of women and men concerning pregnancy, childbirth, and child-rearing. These questions also addressed efforts to prevent unwanted pregnancies and promote health in future pregnancies. Differences were found between Korean male and female adolescents in the awareness of gender equality related to pregnancy, birth, and HPPB [7]. Measuring nursing concepts associated with healthy pregnancies may benefit future research on healthy pregnancies among both adolescents and those in other age groups. This information could provide a foundation for developing guidelines or national policies aimed at healthy pregnancy management for young adolescents.

In Korea, which has the world’s lowest birth rate, pregnancy at older ages has also become more common. Specifically, the proportion of pregnant women aged 35 years or older increased by 13.3% over the past 10 years [11]. This demographic shift has led to many unmarried men and women of childbearing age becoming indifferent to the concept of a healthy pregnancy or passive in their preparations for it. Research focused on unmarried college students has indicated that fostering self-efficacy in pregnancy planning [12], emphasizing the importance of preventive depression management for a healthy pregnancy [13], and increasing the perceived value of motherhood and fatherhood could help promote childbirth [14]. The theoretical frameworks and measurement tools employed in these studies [7,12-14] may be instrumental in tailoring education to meet the needs of unmarried Korean adults regarding healthy pregnancies. They also suggest parameters for nursing research in this area.

## Perspective 2

### Social determinants of health perspective: expanding views on healthy pregnancies, high-risk pregnancies, and the nursing role

Social determinants of health highlight the various conditions and environments in which people are born, live, learn, work, play, worship, and age. These factors influence a range of health and functional outcomes, along with quality of life. Social determinants of health can be categorized into five domains: (1) economic stability, (2) education access and quality, (3) healthcare access and quality, (4) the neighborhood and built environment, and (5) the social and community context [15]. Since healthy pregnancies are influenced by the interplay of these factors, both directly and indirectly, disparities and inequalities are evident in real-world scenarios. Recognizing that individuals, families, workplaces, cultures, and society play roles in supporting healthy pregnancies, social determinants of health serve as a framework for nurses and nurse researchers to critically examine the factors associated with healthy pregnancies from a multifaceted, high-level perspective.

#### 1) Expanding social awareness of healthy pregnancies

A recent article discussed the roles of men and women at the individual, family, and societal levels concerning the public perception of healthy pregnancies and specific efforts to prevent high-risk pregnancies [16]. The study highlighted the importance of raising awareness that healthy pregnancies are a social and cultural issue of common interest across generations, while underscoring the responsibility of the nursing field in this endeavor.

Within the framework of the HP4ALL project [9], these factors could be instrumental in investigating current and potential nursing issues, as well as in pursuing new avenues for research on healthy pregnancies, including (1) the environment, with a focus on interventions in the local community and the external environment; (2) targeted populations, considering predisposing factors, enabling risks, and needs; and (3) outcomes such as the utilization of PCC services and behavioral changes related to PCC risk factors.

#### 2) Identifying current and potential nursing clients who face inequality, discrimination, and alienation and providing them with professional nursing care

If we acknowledge the right to a healthy pregnancy and childbirth, we must be attentive to those whose rights are compromised or who face discrimination. The nursing needs of marginalized or disadvantaged groups must be recognized and addressed. For instance, non-obstetric hospital wards may include vulnerable patients or care recipients who face current or potential risks for pregnancy and childbirth complications. Nursing interventions therefore must be developed to identify and meet their common needs, while considering the unique aspects of pregnancy and childbirth. For instance, the discriminatory structures and cultures faced by individuals with disabilities underscore the necessity for social support measures that protect maternal rights during pregnancy and childbirth [17]. Additionally, a multidisciplinary approach is currently being employed to tailor prepregnancy counseling recommendations for patients with inflammatory bowel disease, a common chronic condition [18]. Furthermore, individualized management plans for women diagnosed with cancer during pregnancy, as well as their families, have been formulated by care teams that include nurses [19]. The development of specialized professional nursing care for these vulnerable clients can promote healthy pregnancies among marginalized individuals and groups.

## Conclusion

Nursing plays a pivotal role in safeguarding a universal right to health, with a foundational step being the promotion of healthy pregnancies. In an era characterized by low birth rates, the role of nursing in promoting health in the context of pregnancy has become increasingly critical.

Customized nursing care that embraces gender equality and a life cycle approach, while also integrating a social determinants of health perspective, can enrich the body of nursing knowledge concerning healthy pregnancy. Additionally, this approach could promote the development of diverse and innovative nursing interventions. Ultimately, such advancements would broaden the scope of nursing’s role in fulfilling its social responsibility to promote healthy pregnancies.
